# Flow Experience and Innovative Behavior of University Teachers: Model Development and Empirical Testing

**DOI:** 10.3390/bs15030363

**Published:** 2025-03-14

**Authors:** Xing Chen, Ling Wu, Lehan Jia, Mohammed A. M. AlGerafi

**Affiliations:** 1 College of Education, Zhejiang Normal University, Jinhua 321004, China; 2 School of Foreign Languages, Zhejiang Gongshang University, Hangzhou 310018, China

**Keywords:** university teachers, flow experience, innovative behavior, SOR theory, structural equation modeling

## Abstract

The innovative behavior of university teachers plays a vital, long-term role in advancing scientific and technological innovation and in nurturing high-level talent. Flow experience is influenced by flow antecedents such as the balance between challenge and skill, clear goals, immediate feedback, intrinsic motivation, and perceived risk. Moreover, flow experience, characterized by deep concentration and effective attention allocation, is essential in facilitating innovative behavior by enhancing problem-solving and analytical abilities. This study explores the relationship between flow experience and innovative behavior among university teachers, providing a fresh theoretical perspective for encouraging such behavior. To investigate this, the study developed the “University Teacher Flow Experience Scale” and the “University Teacher Innovative Behavior Scale”. A survey of 316 university teachers in China was conducted, with statistical analysis utilizing variance analysis and structural equation modeling. Results showed that both flow experience and innovative behavior were at moderate levels. Significant variations in innovation levels were noted across disciplines, professional titles, and positions, but no gender differences were found. Antecedents such as a balance between challenges and skills, clear goals, immediate feedback, and intrinsic motivation positively influenced flow experience, while perceived risk had a negative impact. Flow experience itself significantly enhanced innovative behavior among university teachers. The findings highlight the importance of optimizing the factors contributing to flow experience at institutional and individual levels to promote innovation in higher education.

## 1. Introduction

University faculty members constitute a pivotal force not only in scientific and technological innovation but also in nurturing top-tier innovative talents. Stimulating faculty innovation plays a fundamental, long-term, and essential role in advancing scientific and technological innovation and cultivating such talents. Existing research primarily views teacher innovation behavior as a spontaneous and creative behavior encompassing opportunity exploration, idea generation, and concept realization (Messmann & Mulder, 2014). It advocates tapping into teachers’ innovative potential, fostering their creative thinking and creativity, and improving the conditions and environment for innovation by focusing on individual factors such as personality traits and thinking modes, organizational factors including resource integration, leadership styles, and institutional cultures, and social factors like social capital, social support, and social networks (Yan, 2023). Psychologist Mihaly Csikszentmihalyi, through in-depth interviews with 91 globally prominent and creative individuals across natural scientists, artists, and writers (including 14 Nobel laureates), found that individual creativity is closely related to flow experiences. Individuals who experience more flow states of total concentration and self-forgetfulness typically demonstrate higher creativity (Csikszentmihalyi, 1997). Furthermore, flow experiences not only elicit pleasure but also serve as a motivator for corporate employees to fully engage in their work, ultimately enhancing innovative behavior (Maqbool et al., 2019; Peng et al., 2024).

## 2. Literature Review

**Figure 1:**
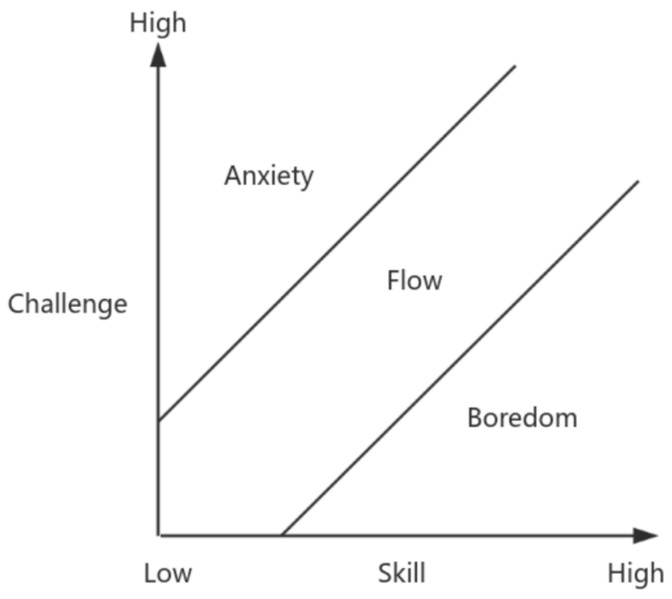
Three-channel model.

**Figure 2:**
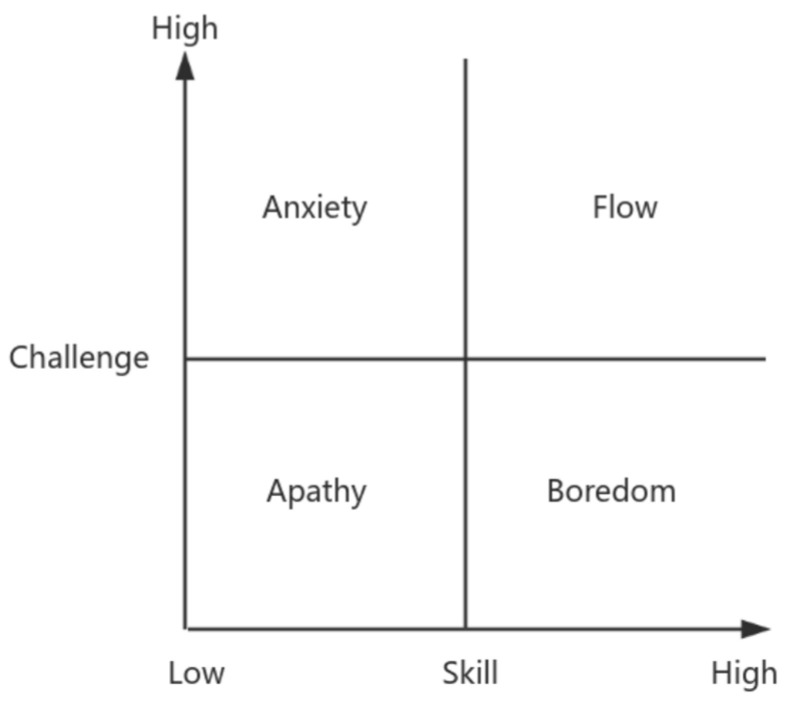
Four-channel model.

Flow is generally recognized as a positive and intrinsically motivating state of consciousness, embodying an optimal condition that is associated with positive emotions, motivations, and cognitive experiences. Flow arises from deep engagement in an activity and signifies an optimal experience of intrinsic reward (Csikszentmihalyi, 2000). When an individual attains a state of flow, they exhibit heightened concentration on the task at hand, a seamless integration of knowledge and action, a profound sense of absorption and self-forgetfulness, an accelerated perception of time passing, and a harmonious fusion of behavior and consciousness, all of which collectively contribute to the experience of pleasure and a profound sense of meaning. Previous research has examined numerous factors that influence the flow experience. Regarding the antecedents of flow, pertinent theories encompass the three-channel model (refer to Figure 1), the four-channel model (refer to Figure 2) and the PAT model, which primarily incorporate factors such as challenge–skill balance, clear goals, immediate feedback, intrinsic motivation, and perceived risk. Factors including challenge–skill balance, clear goals, and immediate feedback influence individuals’ flow experience by affecting their level of attention concentration, sense of control (Keller & Blomann, 2008), and interactive behavior. This has been corroborated in contexts such as internet usage (Trevino & Webster, 1992), online shopping experiences (Novak et al., 2000), and web utilization (Skadberg & Kimmel, 2004). Conversely, individual factors such as intrinsic motivation, perceived risk, and personal traits influence the flow experience by impacting individuals’ curiosity (Schutte & Malouff, 2020; Sofyan et al., 2024), intrinsic interests, enthusiasm for work (Wang, 2022), levels of engagement, and degree of control (Chou & Ting, 2003). This has been exemplified in areas like gaming user experience (Hung et al., 2015), user-generated content (Moon et al., 2014), and online learning platform user experience (Mulik et al., 2020).

From the perspective of flow consequences, flow exerts a significant impact on individuals’ cognition, emotions, and behaviors, with a particularly notable effect on fostering innovative behavior. The flow experience helps individuals enhance the novelty and creativity of their thoughts and behaviors, thereby promoting innovative behavior, by increasing neural activity in specific brain regions (David et al., 2024), enhancing concentration, promoting rational allocation of attention (Yoshida et al., 2014), refining problem-solving and analytical skills (Vogt, 2005), bolstering intrinsic motivation and engagement, and amplifying feelings of pleasure (Huang et al., 2018), fulfillment (P. Lin, 2023), and well-being (Tsaur et al., 2013). This has been extensively validated across sectors such as the service industry (Kafeel et al., 2024), cultural and creative industries (Dai et al., 2024), user-driven music collaborations (Xu et al., 2023), software development (Zubair & Kamal, 2015), and the cultivation of undergraduate nursing students (Y. Lin et al., 2025).

This study endeavors to apply flow theory to the field of education, exploring the various factors influencing the emergence of flow experiences among university teachers and the influence of these flow experiences on the enhancement of their innovative behaviors. Drawing on the Stimulus–Organism–Response (SOR) theory, this study constructs a model for the generation of flow experiences and the fostering of innovative behaviors among university teachers. It focuses on exploring institutional transformation pathways that promote the generation of flow experiences and the cultivation of innovative behaviors among university teachers, thereby offering a new theoretical perspective and practical countermeasures for the improvement of innovative behaviors in the higher education sector.

## 3. Theoretical Framework and Hypotheses

### 3.1. Antecedents of Flow

Flow is a psychological state akin to being “enraptured”, characterized by a profound integration of individual consciousness, cognition, abilities, and behaviors. It typically manifests in features such as heightened concentration (full immersion), loss of self-awareness (absorption in the task), and time distortion (perception of time slowing down or speeding up). By improving focus, strengthening intrinsic motivation, optimizing decision-making abilities, reinforcing feelings of accomplishment, enhancing mental health, and stimulating innovative thinking, flow can enhance personal work efficiency, creativity, and well-being. In the context of university teachers’ work, flow experiences hold considerable potential and directly influence their innovative behaviors. However, due to factors such as the nature of work, individual characteristics, and educational systems, university teachers’ flow experiences are subject to certain disturbances or limitations. Consequently, their innovative behaviors are not effectively stimulated or fully unleashed, leading them into a vicious cycle characterized by “low flow experiences–low innovative behaviors”.

Drawing on a comprehensive review of both domestic and international research, the emergence of flow experiences is primarily influenced by the following five key antecedent factors:

(1) Challenge–Skill Balance: This balance refers to the alignment between the complexity of tasks or activities and an individual’s skill level, which is crucial for achieving optimal psychological states. According to the four-channel model of flow, the matching of challenge and skill can roughly form four types of psychological states: flow (high challenge and high skill), boredom or relaxation (low challenge and high skill), apathy (low challenge and low skill), and anxiety (high challenge and low skill). When tasks are either too simple or too difficult relative to an individual’s skill level, it becomes difficult for them to experience flow. Flow experiences are most likely to occur when challenge and skill are near a dynamic balance at a higher level (Larche & Dixon, 2020). Many university teachers struggle to achieve work-related flow due to several factors. On one hand, they perceive their teaching, research, and management skills as inadequate to address the escalating challenges of scientific research, title promotion, performance evaluations, tenure assessments, and award competitions, resulting in persistent high stress and anxiety. On the other hand, they often find routine tasks such as classroom teaching, financial reimbursement, and student management lack sufficient challenge, leading to boredom and burnout. Based on this analysis, the study proposes the following hypothesis:

**Hypothesis** **1.** *The challenge–skill balance significantly affects the flow experiences of university teachers.*

(2) Clear Goals: Clear goals entail an individual’s precise cognition and delineation of intended actions, task content, and anticipated outcomes before or during an activity, alongside the accurate identification and assessment of these goals. By guiding individuals to maintain a heightened level of concentration on tasks, clear goals enable the effective filtering of information or distractions unrelated to the current task (Erhel & Jamet, 2019), thereby minimizing the dispersion and wastage of cognitive resources and facilitating the spontaneous emergence of the flow (Cheng, 2024). University teachers exhibit varying degrees of goal absence in both teaching and research endeavors. Constrained by factors such as teaching experience, curriculum design, and individual differences among students, universities find it challenging to establish clear and quantifiable goals for the effectiveness of knowledge transmission and student competency enhancement in teaching, which hinders teachers from experiencing flow in their teaching work. Scientific research constitutes creative endeavors aimed at exploring the unknown, advancing theories, or innovating technologies, with related goals often being relatively vague, abstract, multidimensional, and dynamically evolving. In their research endeavors, university teachers frequently encounter difficulties in identifying valuable and feasible research goals within a specific timeframe. Performance evaluations further exacerbate the short-term and volatile nature of these research goals, posing challenges for teachers to swiftly focus their attention and systematically formulate research goals over the long term. Consequently, this hinders their capacity to achieve optimal flow experiences. Based on this analysis, the study proposes the following hypothesis:

**Hypothesis** **2.** *Clear goals have a significant impact on the flow experiences of university teachers.*

(3) Immediate Feedback: Immediate feedback pertains to an individual’s capacity to receive information swiftly and precisely regarding their current performance or task progress during an activity. This feedback can stem from endogenous sources, rooted in the individual’s own perceptions, sensations, and reflections (e.g., self-assessment and sensory experiences), or exogenous sources, originating from the external environment or direct feedback from others (e.g., peer reactions, organizational evaluations, and system data). Immediate feedback not only assists individuals in self-monitoring and adjustment, enabling them to evaluate their performance, strategy implementation, and the disparity between current progress and established goals in a timely manner (Peifer et al., 2020), but also effectively alleviates cognitive load, fortifies the linkage between behavior and outcomes, augments the sense of control over actions, and fosters the emergence of flow experiences (Zhang et al., 2023b). The outcomes and benefits associated with education and teaching, talent cultivation, and scientific research are notably ambiguous and delayed, posing challenges for university teachers in obtaining clear and immediate exogenous feedback in their work. This impediment hinders their capacity to promptly adjust their development direction and action strategies, thereby weakening their intrinsic connection to their work and making it difficult to attain a state of flow. Additionally, university teachers, constrained by school management systems, tend to rely on exogenous information characterized by greater variability and delay, neglecting endogenous feedback, which further obstructs the generation of flow experiences. Based on this analysis, the study proposes the following hypothesis:

**Hypothesis** **3.** *Immediate feedback has a significant impact on the flow experiences of university teachers.*

(4) Intrinsic Motivation: Intrinsic motivation refers to the tendency to engage in an activity autonomously, driven by factors such as interest, aspirations, passion, enjoyment, and the pursuit of achievement, without relying on external rewards or pressures. The stronger an individual’s intrinsic motivation for a particular activity, the easier it is for them to enter a state of flow (Waterman et al., 2003). Without the support of intrinsic motivation, it becomes difficult for an individual to achieve the deep immersion and high concentration required for flow over an extended period within an activity (Csikszentmihalyi & LeFevre, 1989). Driven by factors such as the administrative orientation of universities, the industrialization of education, and the industrialization of academic production systems, some university teachers’ original intrinsic motivations, such as their sense of mission, curiosity, and “pursuit of academics as a career”, may be partially overshadowed by external goals such as money, career advancement, social status, and honorary awards. This results in the loss of important conditions for entering a state of flow. Based on this analysis, the study proposes the following hypothesis:

**Hypothesis** **4.** *Intrinsic motivation significantly influences the flow experiences of university teachers.*

(5) Perceived Risk: Perceived risk refers to an individual’s intuitive judgment and psychological reaction to the uncertainty of event outcomes and the severity of potential adverse consequences. When an individual’s perceived risk is high, it continuously occupies their attentional resources, affecting their sense of engagement, concentration, and pleasure, thereby hindering their ability to enter a state of flow (Chen et al., 2017). University teachers face considerable uncertainties in areas such as publishing papers, applying for research grants, recruiting, and training graduate students, and evaluating professional titles. Institutional factors such as the “up-or-out” policy, last-rank elimination, pre-tenure systems, thesis reviews, employment rate assessments, and performance evaluations further heighten their perceived risk of adverse outcomes, which is detrimental to the generation of flow experiences. Based on this analysis, the study proposes the following hypothesis:

**Hypothesis** **5.** *Perceived risk significantly influences the flow experiences of university teachers.*

### 3.2. Flow and Innovative Behavior

Flow experiences have a significant impact on the innovative behaviors of university teachers. On the one hand, individuals in a flow state are at their optimal level in terms of attention allocation, divergent thinking, knowledge integration, problem-solving, and potential activation, making them the most creative. When university teachers enter a flow state, they exhibit highly focused attention, clear, fluent, and active thinking, with their subconscious fully awakened or even dominating. Their imagination, knowledge integration ability, information processing capability, and problem-solving skills are all at their best, which is conducive to the generation of innovative behaviors. On the other hand, flow experiences possess a self-reinforcing mechanism that helps form a virtuous cycle between flow experiences and innovative behaviors (Liu et al., 2023). Flow experiences can produce positive emotions and outcomes such as pleasure, fulfillment, and meaning. University teachers who have experienced flow will actively seek out flow states, increasing their dedication and enthusiasm for their work, and continuously engaging in innovative behaviors. Based on this, the study proposes the following research hypothesis:

**Hypothesis** **6.** *Flow experiences significantly influence the innovative behaviors of university teachers.*

### 3.3. Theoretical Model

Drawing on the SOR theory, this study integrates antecedents of flow, flow experiences, and innovative behaviors into a conceptual framework for understanding the generation of flow experiences and innovative behaviors. The SOR theory, initially proposed by American psychologist Woodworth in 1926 and subsequently refined by Mehrabian in 1974, primarily elucidates the comprehensive pathway through which stimuli in the external environment impact an individual’s internal emotional and psychological states, ultimately influencing their behavioral responses (Mehrabian, 1974). Within this theoretical framework: Stimulus (S) encompasses external environmental factors that captivate an individual’s attention and exert an influence on their psychology, including economic considerations such as promotions and pricing, social dynamics like family influences and policies, as well as specific contextual elements. Organism (O) represents the internal psychological, cognitive, emotional, and experiential changes that an individual undergoes in response to external stimuli. Response (R) signifies the behavioral tendencies—whether approach or avoidance—displayed by an individual under the combined influence of stimuli and internal changes, encompassing attitudes, emotions, and intentions. The SOR theory has been extensively applied to analyze consumer behavior in offline retail settings (Gatautis et al., 2016), online shopping within virtual communities (Yin & Qiu, 2021), human–computer interaction on internet platforms (Zhang et al., 2023a), and other pertinent issues, rendering it highly suitable for investigating the interplay between the environment, individual psychological states, and individual behaviors. Considering this, we propose to consider antecedents of flow as external environmental stimuli, flow experiences as the psychological changes perceived by the organism, and innovative behaviors as the resultant behavioral responses of individuals. By adopting this perspective, we construct a comprehensive system for understanding the generation of flow experiences and innovative behaviors.

**Figure 3:**
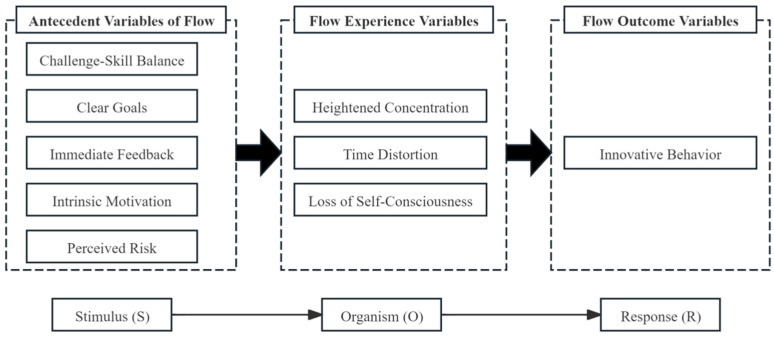
Theoretical model of flow experience and innovative behavior generation among university teachers.

In summary, we have constructed a theoretical framework elucidating the generation of flow experiences and innovative behaviors among university teachers (refer to Figure 3). Firstly, from the perspective of the stimulus phase, the antecedent factors that primarily contribute to the emergence of flow experiences in university teachers encompass challenge–skill balance, clearly defined goals, immediate feedback, intrinsic motivation, and perceived risk. Secondly, regarding organismic transformations, flow is characterized as a work state of heightened attention concentration, self-awareness diminution, and time distortion, which university teachers encounter under the influence of various stimulating factors. Specifically, a more balanced challenge–skill ratio, clearer goals, more immediate feedback, stronger intrinsic motivation, and lower perceived risk in their work result in a higher level of flow experiences among university teachers. Lastly, from the standpoint of response outcomes, innovative behavior emerges as the behavioral manifestation of university teachers who immerse themselves in flow experiences. Consequently, teachers who possess more work-related flow experiences tend to exhibit a greater propensity for generating innovative behaviors.

## 4. Materials and Methods

### 4.1. Date Collection and Sample

Utilizing stratified sampling based on university types, we selected two institutions from each category—namely, ‘Double First-Class’ universities, local universities, and vocational colleges—across three locations within the Yangtze River Delta region, totaling six universities. Seventy questionnaires were administered to each institution, resulting in a total distribution of 420 questionnaires. We received 346 responses, of which 316 were deemed valid, yielding an effective response rate of 91.33%. A total of 420 university teachers employed by these institutions received the questionnaire via their work email. In addition to demographic questions, the questionnaire encompassed assessments of flow antecedents such as the balance between challenges and skills, clear goals, and immediate feedback; flow experience factors; and measurements of innovative behavior. Accompanying the questionnaire was an explanation outlining the purpose of the study, which aimed to explore “innovative behaviors among university teachers”, and assured all participants of their right to informed consent and the freedom to withdraw from the survey at any time.

The demographic descriptive statistics of the sample, which totaled 316 teachers, revealed that there were 139 male teachers (44.0%) and 177 female teachers (56.0%); 122 teachers were in natural sciences (38.6%), 117 in social sciences (37.0%), and 77 in humanities (24.4%); 137 held the rank of lecturer (43.4%), 97 were associate professors (30.7%), and 82 were professors (25.9%).

### 4.2. Measurement

#### 4.2.1. Antecedent Variables of Flow

The antecedent variables encompass challenge–skill balance, clear goals, immediate feedback, intrinsic motivation, and perceived risk. Drawing on “The Flow State Scale” developed by Jackson and Marsh (1996), we assessed the levels of these antecedent variables through items such as “Faced with the complexity of my work, I believe I have the ability to cope”, “When working, I clearly know what I need to achieve”, “I am well aware of my performance at work”, “I believe that the work of a university teacher holds great value”, and “I think it is highly likely that I will not meet my work assessment requirements”. Each dimension comprised three items, measured using a five-point Likert scale, with options ranging from 1 (completely disagree) to 5 (completely agree).

#### 4.2.2. Flow Experience Variables

Based on the research on flow experience (Kawabata et al., 2008), we measured the level of flow experience among university teachers by assessing the extent to which they experienced intense concentration, loss of self-consciousness, and distortion of time while working. This was achieved through items such as “I can often concentrate fully on the task I am working on”, “When working, I don’t feel my own existence”, and “When working, I feel like time passes quickly”. Each dimension consisted of three items, measured using a five-point Likert scale, with options ranging from 1 (completely disagree) to 5 (completely agree).

#### 4.2.3. Flow Outcome Variables: Innovative Behavior of University Teachers

Drawing on the “Employee Innovative Behavior Scale” (Scott & Bruce, 1994), we assessed the innovative behavior of university teachers using four items: “I am adept at identifying new problems and actively engage with new fields, phenomena, and directions”, “I frequently generate new ideas and am capable of proposing new theories, hypotheses, and perspectives”, “I enjoy exploring new methods and innovative ways to solve problems”, and “I am capable of proposing new solutions and creatively interpreting or addressing problems”. These items were measured using a five-point Likert scale, with options ranging from 1 (completely disagree) to 5 (completely agree).

#### 4.2.4. Control Variables

Consistent with relevant research, we selected gender, discipline, professional title, and job position as control variables. Table 1 presents the variables and their definitions.

#### 4.2.5. Questionnaire Reliability and Validity Test

Confirmatory factor analysis was conducted using AMOS 26.0 to assess the item reliability, composite reliability, convergent validity, and discriminant validity of the questionnaire. As shown in Table 2, the results revealed that all items of the questionnaire indicators had standardized loadings (Std) greater than 0.6, squared multiple correlations (SMC) greater than 0.3, composite reliability (CR) values greater than 0.8, Cronbach’s α coefficients greater than 0.8, and average variance extracted (AVE) values greater than 0.5. As shown in Table 3, the square root of the AVE for each factor was greater than its correlation coefficients with other factors (the diagonal numbers in Table 3 represent the square roots of the AVE, while the other values represent the correlation coefficients). These results indicate that the questionnaire has good reliability and validity, supporting the subsequent structural equation modeling analysis.

#### 4.2.6. Data Analysis

Statistical analysis of the data was conducted using SPSS 23.0 in this study. Firstly, means and standard deviations were used to evaluate the levels of various continuous variables. Secondly, independent sample t-tests and one-way ANOVA tests were employed to analyze the differences in university teachers’ innovative behavior based on gender, discipline, position, and professional title. Finally, AMOS 26.0 was utilized for path analysis of the structural equation model to examine the pathways through which flow experience influences innovative behavior.

## 5. Results

### 5.1. Descriptive Statistics

As shown in Table 4, the descriptive statistics indicate that the mean score for university teachers’ innovative behavior is 2.50, suggesting a moderately low level and highlighting considerable potential for enhancement in this domain. The mean score for university teachers’ flow experience is 2.72 on a five-point scale, indicating a moderate level. Specifically, the mean scores for loss of self-consciousness, high concentration of attention, and time distortion are 2.73, 2.70, and 2.69, respectively. These findings underscore the urgent need to improve the level of flow experience among university teachers in their work.

### 5.2. Analysis of Differences

As shown in Table 5, the differences in the levels of innovative behavior among university teachers across various control variables are as follows: (1) No significant gender difference is observed in the innovative behavior of university teachers, with male teachers (M = 2.54) scoring slightly higher than female teachers (M = 2.48). (2) Significant differences are observed in innovative behavior across disciplines. Specifically, university teachers in natural sciences exhibit the highest level of innovative behavior (M = 2.90), followed by those in social sciences (M = 2.33), and the lowest in humanities (M = 2.14). These findings suggest that natural sciences, which primarily focus on objective laws and the creation and application of new technologies, are more conducive to generating innovative behavior compared to social sciences and humanities that deal with complex social phenomena requiring new interpretations and theories. (3) Significant differences in innovative behavior are also found across professional titles. Lecturers have the highest level of innovation (M = 2.75), followed by associate professors (M = 2.39), and professors have the lowest (M = 2.23). This indicates that lecturers and associate professors, who are in the ascending phase of their career development and in their prime years, possess stronger career development motivation, higher enthusiasm for scientific research, and more energy, making them more likely to engage in innovative behavior. In contrast, professors, who are in the stable phase of their career development and may experience declines in health and energy levels, tend to reduce their workload, resulting in a decrease in innovative behavior. (4) Significant differences in innovative behavior are also evident based on administrative positions. University teachers who do not hold administrative positions (M = 2.80) demonstrate significantly higher levels of innovative behavior than those who do (M = 2.30). This suggests that university teachers with administrative responsibilities often face more complex and trivial administrative tasks, which divert their time and energy, making it difficult for them to engage in innovative behavior. On the other hand, university teachers without administrative duties have relatively fixed job responsibilities, allowing them to concentrate better on deep research in a specific field, thereby facilitating innovative behavior.

### 5.3. Path Analysis of Structural Equation Modeling

**Figure 4:**
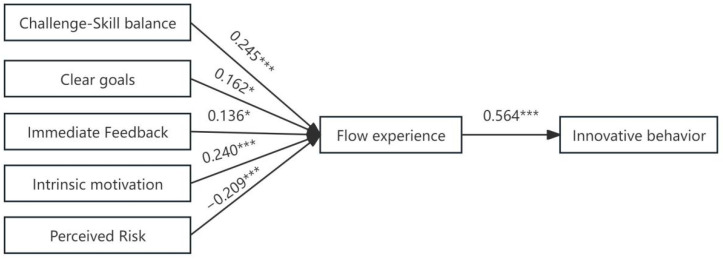
Results of the analysis of influence paths between flow experience and innovative behavior among university teachers. Note: * *p* < 0.05, *** *p* < 0.001.

Based on the flow experience and innovative behavior generation model of university teachers, a structural equation model was constructed. The goodness-of-fit of the model was tested (results are shown in Table 6), and the model path coefficients were analyzed (results are presented in Table 7 and Figure 4). The model fit indices were as follows: χ^2^/df = 1.698, RESEA = 0.047, GFI = 0.882, IFI = 0.950, and CFI = 0.950, all falling within acceptable ranges, indicating a good fit of the model. In Table 6, Estimate and Std represent the unstandardized and standardized path coefficients, respectively, with all paths being significant at *p* < 0.05.

At the antecedent level, challenge–skill balance (β = 0.245, *p* < 0.001), clear goals (β = 0.162, *p* < 0.05), immediate feedback (β = 0.136, *p* < 0.05), and intrinsic motivation (β = 0.240, *p* < 0.001) all significantly positively influenced the flow experience of university teachers. Furthermore, the challenge–skill balance had the greatest impact on university teachers’ flow experience, followed by intrinsic motivation and clear goals, with immediate feedback having the least impact. The Hypotheses 1, 2, 3, and 4 were thus verified. Perceived risk (β = −0.209, *p* < 0.01) significantly negatively influenced the flow experience of university teachers, confirming Hypothesis 5. At the outcome level, flow experience (β = 0.564, *p* < 0.001) significantly positively influenced the innovative behavior of university teachers, suggesting that teachers with more flow experience generated more innovative behaviors. Thus, Hypothesis 6 was also verified.

## 6. Discussion and Implications

### 6.1. Discussion

The research results indicate that the challenge–skill balance, clear goals, immediate feedback, and intrinsic motivation all exert significant positive effects on the flow experience of university teachers. The results of this study are in accordance with the findings reported by Jin (2012) and the research undertaken by Yen and Lin (2022). Conversely, perceived risk has a significant negative impact on their flow experience, which is consistent with the findings of PHAM (2024).

The influence of the challenge–skill balance, clear goals, immediate feedback, and intrinsic motivation on the flow experience of university teachers can be explained from two perspectives: the university management system and the job characteristics of university teachers. From the perspective of the university management system, the current evaluation system for scientific research in universities emphasizes quantitative evaluation, performance appraisal, and intense competition, creating strong pressure on university teachers regarding their survival and development. This leads to an imbalance between work challenges and their skills, with intrinsic motivation being crowded out by extrinsic motivation. The perceived risks, such as economic losses and career development crises, increase, leading to negative emotions like anxiety and tension, which hinder the occurrence of flow experience. From the perspective of job characteristics, the work of university teachers, including teaching, research, and social service, is inherently complex, uncertain, and creative. Work goals, particularly research objectives, can be somewhat ambiguous, making it challenging to immediately guide teachers into a state of flow. Additionally, the difficulty in precisely measuring the achievements of teaching, research, and other tasks, coupled with individuals’ tendency to rely on external evaluation information, results in a certain degree of lag in work feedback. Furthermore, the creative work involved in exploring new theories, technologies, and viewpoints often has a long cycle, contrasting with the clear indicators and immediate feedback associated with technical work. This further exacerbates the lack of flow experience among university teachers. Consequently, those who cannot tolerate the vagueness of long-term goals and the lag in feedback may resort to “short, frequent, and quick” research methods, leading to a dual absence of flow experience and innovative behavior.

Flow experience is positively correlated with innovative behavior among university teachers, echoing the research conclusions of Khan (Khan et al., 2021). Teachers who experience more flow are more likely to exhibit innovative behavior. Flow experience expands their attention, cognition, and action scope; fosters positive psychological resources; broadens their “instantaneous thought-action repertoire”; and prompts them to explore new possibilities and act, thereby nurturing innovative behavior in their work. Moreover, flow can catalyze the second-order growth of innovative behavior. When university teachers engage in work-related flow activities, they are more likely to invest additional time and energy in their work, increasing the likelihood of generating innovative behavior and establishing a virtuous cycle of high flow experience and high innovative behavior.

This study proposes suggestions for promoting flow experience and fostering innovative behavior among university teachers from two perspectives: school management and institutional reform, as well as cognitive and behavioral adjustments for individual teachers. In terms of fostering a balance between challenges and skills, universities should establish a differentiated evaluation system to promote a high-level dynamic balance between teachers’ work challenges and skill levels. Teachers can assess and adjust the alignment between challenges and skills based on the four-channel model of flow, avoiding the boredom of low skill–low challenge and the anxiety of low skill–high challenge situations (Csikszentmihalyi, 1988). Regarding the improvement of immediate feedback, universities should strengthen process-based evaluations by constructing a multi-subject, multi-step, and multi-channel information collection and processing system, providing timely external feedback on teachers’ work progress and achievements. Teachers should establish personalized self-assessment and feedback frameworks based on educational goals, scientific spirit, and professional ethics, regularly engaging in self-reflection and assessment to actively avoid the interference of external utilitarian feedback. In terms of enhancing intrinsic motivation, universities should pursue a balance between instrumental rationality and value rationality, strengthening teachers’ sense of responsibility and mission, and guiding them to teach with dedication, student-centeredness, and a commitment to academia, thereby effectively protecting teachers’ intrinsic motivation. Teachers should maintain educational enthusiasm and a spirit of exploration and choose fields that stimulate their curiosity and have inherent attractiveness to them, moving away from the misconception of valuing achievements over processes. To reduce perceived risks, universities should seek a balance between pressure and motivation, establishing a relatively free, open, and tolerant inclusive education system that provides teachers with a certain degree of room for trial and error, along with relevant risk-sharing mechanisms. Teachers should actively adjust cognitive misconceptions, revise overestimations of risk probability and losses, and enhance their risk prevention and control capabilities. In terms of constructing a linkage mechanism between flow and innovative behavior, universities should strengthen the monitoring of teachers’ flow experiences, establishing a comprehensive flow generation support system for teacher teams seeking significant innovations, and building multidimensional linkage channels between teachers’ flow experiences and innovative behaviors. Teachers should allocate the time when they are most likely to experience flow to more creative work, creating a workspace conducive to concentration and the stimulation of creative thinking, thereby providing fertile ground for the generation of flow experiences and innovative behaviors.

### 6.2. Theoretical Contributions

Firstly, this study is among the early ones to introduce the research on flow theory in relation to innovative behavior into the analysis of educational issues, highlighting that enhancing flow experiences serves as a novel key to stimulating innovative behavior among university teachers.

Secondly, previous studies have shown that individuals are more likely to experience flow in the workplace (Gomezel & Aleksić, 2020), and moreover, that flow has a positive effect on enhancing individual work capabilities. However, the intricate linkage between flow experiences and the emergence of innovative behavior remains an area of ambiguity. The present study advances the comprehension of the underlying mechanisms that govern flow experiences and the generation of innovative behavior by establishing a theoretical framework that incorporates factors such as challenge–skill balance, definitive goals, prompt feedback, intrinsic motivation, and perceived risk.

### 6.3. Practical Implications

Firstly, at the level of management policy optimization, amidst the dual predicaments of high costs and low efficiency faced by traditional rigid educational reforms, a flexible innovation promotion system centered on cognitive intervention strategies has been established. This system integrates micro-intervention mechanisms for cultivating university teachers’ flow experience, including balancing challenges with skills, reducing short-term utilitarian goals, constructing multi-channel feedback mechanisms, enhancing teachers’ sense of responsibility and mission, establishing a relatively free, open, and tolerant inclusive educational institutional system, and strengthening the monitoring of teachers’ flow experiences. These mechanisms provide university administrators with a practical policy toolbox, effectively bridging the implementation gap between macro-institutional design and individual behavior change.

Secondly, at the level of individual teacher behavior adjustment, an innovative micro-individual intervention path for flow-innovation behavior has been developed. By implementing subtle “nudges” such as challenge–skill assessments, career goal optimization and adjustment, self-feedback mechanism construction, intrinsic motivation enhancement, risk prevention and control capability improvement, and the linkage mechanism between flow and innovation behavior, teachers are assisted in overcoming innovation barriers within the existing institutional framework. This facilitates a cognitive leap from passive adaptation to active creation, providing psychological momentum for sustained innovation among teachers.

### 6.4. Limitations and Future Directions

Firstly, this study is cross-sectional research. Despite its comprehensive theoretical analysis and detailed empirical testing, utilizing high-reliability and high-validity measurement tools for data analysis, cross-sectional studies are unable to uncover the longitudinal dynamic changes and long-term mechanisms between variables. Therefore, future research should select representative university teachers from various career stages for longitudinal follow-up surveys to further enhance the reliability of the research conclusions.

Secondly, this study only considers the direct mechanism between flow experience and teachers’ innovative behavior, without incorporating external factors such as university management systems and organizational culture that may influence university teachers’ flow experience and innovative behavior. Future research may need to focus on external institutional variables and further explore the interaction between institutions, flow experience, and innovative behavior.

Lastly, the sample size of the survey data in this study is relatively small, which may not fully reflect the diversity and complexity of the impact of university teachers’ flow experience on their innovative behavior. Subsequent research will further expand the sample size to enhance the generality and representativeness of the research conclusions.

## 7. Conclusions

This study develops a model to examine the influencing factors of flow experience and innovative behavior among university teachers, grounded in the SOR framework and utilizing data collected from 316 survey questionnaires. The findings reveal that a balance between challenge and skill, clear goals, immediate feedback, and intrinsic motivation exerts significant positive influences on university teachers’ flow experience, whereas perceived risk has a notable negative impact. Moreover, flow experience is found to affect university teachers’ innovative behavior significantly positively. Additionally, this study suggests strategies for optimizing the antecedents of flow experience from two dimensions: institutional management and policy, as well as individual teachers’ cognitive and behavioral adaptations. These strategies aim to foster the development of flow experience and innovative behavior among university teachers and offer insights into shifting management paradigms from rigid institutional controls to flexible psychological guidance in enhancing teachers’ innovative behaviors.

## Figures and Tables

**Table 1:** Variables and their definitions.

Variable Type	Variable Name	Description
Antecedent Variables of Flow	Challenge–Skill Balance	Degree of match between job complexity and skills; degree of match between job creativity and skills
	Clear Goals	Clearly know what is wanted; clearly know what to do; clearly know to what extent to achieve
	Immediate Feedback	Immediate understanding of job performance; immediate understanding of job progress; immediate understanding of job effectiveness
	Intrinsic Motivation	Recognition of job value; recognition of job interest; recognition of job achievement
	Perceived Risk	Uncertainty of job outcomes; likelihood of risk occurrence; magnitude of risk loss
Flow Experience Variables	High Concentration of Attention	Duration of high concentration of attention; intensity of high concentration of attention; frequency of high concentration of attention
	Time Distortion	Feeling time passes faster; feeling time passes slower; not feeling the passage of time
	Loss of Self-Consciousness	Weakening of self-awareness; not feeling one’s own existence; merging of action and awareness
Flow Outcome Variables	Innovative Behavior	Discovering new problems; generating new ideas; seeking new methods; proposing new solutions
Control Variables	Gender	Male = 1; Female = 2
	Discipline	Natural Sciences = 1; Social Sciences = 2; Humanities = 3
	Academic Title	Lecturer = 1; Associate Professor = 2; Professor = 3
	Position	Yes = 1; No = 2

**Table 2:** Reliability and convergent validity test results.

Variable	Std	SMC	AVE	CR	Cronbach’s α
Flow Experience	0.692~0.752	0.491~0.566	0.516	0.905	0.905
Challenge–Skill Balance	0.738~0.829	0.545~0.687	0.607	0.822	0.821
Clear Goals	0.773~0.831	0.598~0.691	0.644	0.844	0.844
Immediate Feedback	0.776~0.829	0.602~0.687	0.647	0.846	0.845
Intrinsic Motivation	0.778~0.816	0.605~0.666	0.643	0.844	0.843
Perceived Risk	0.782~0.820	0.612~0.672	0.637	0.840	0.840
Innovative Behavior	0.724~0.792	0.524~0.627	0.571	0.842	0.842

**Table 3:** Discriminant validity test results.

	1	2	3	4	5	6	7
1. Innovative Behavior	**0.756**						
2. Intrinsic Motivation	0.515	**0.798**					
3. Perceived Risk	−0.483	−0.503	**0.802**				
4. Immediate Feedback	0.483	0.542	−0.573	**0.804**			
5. Clear Goals	0.491	0.532	−0.572	0.517	**0.802**		
6. Challenge–Skill Balance	0.462	0.522	−0.466	0.542	0.582	**0.779**	
7. Flow Experience	0.530	0.620	−0.603	0.591	0.610	0.627	**0.718**

Note: the bold numbers along the diagonal of the table represent the square root values of AVE, and the lower triangle represents the Pearson correlation coefficients of the constructs.

**Table 4:** Results of descriptive statistics.

	Min	Max	Mean	Standard Deviation
Innovative Behavior	1	5	2.50	0.96
Flow Experience	1	5	2.72	0.93
High Concentration of Attention	1	5	2.70	0.97
Time Distortion	1	5	2.69	0.98
Loss of Self-Consciousness	1	5	2.73	1.01

**Table 5:** Results of difference analysis.

Variable	Group	Innovative Behavior M ± SD	T/F Value
Gender	Male	2.54 ± 1.07	0.558
	Female	2.48 ± 0.86	
Discipline	Natural Sciences	2.90 ± 1.16	19.781 ***
	Social Sciences	2.33 ± 0.77	
	Humanities	2.14 ± 0.57	
Academic Title	Lecturer	2.75 ± 1.12	9.192 ***
	Associate Professor	2.39 ± 0.82	
	Professor	2.23 ± 0.67	
Administrative Position	Yes	2.30 ± 0.76	−4.498 ***
	No	2.80 ± 1.12	

Note: *** *p* < 0.001.

**Table 6:** Model fit test results.

	χ^2^	DF	χ^2^/DF	RESEA	GFI	IFI	CFI
Measured Value	567.277	334	1.698	0.047	0.882	0.950	0.950
Compliance Status			YES	YES	YES	YES	YES

**Table 7:** Test results of path coefficient of structural equation model.

IV→DV	Estimate	S.E.	C.R.	*p*	Std
Challenge–Skill Balance→Flow Experience	0.202	0.056	3.614	***	0.245
Clear Goals→Flow Experience	0.124	0.053	2.351	0.019 *	0.162
Immediate Feedback→Flow Experience	0.111	0.054	2.032	0.042 *	0.136
Intrinsic Motivation→Flow Experience	0.193	0.053	3.684	***	0.240
Perceived Risk→Flow Experience	−0.159	0.051	−3.137	0.002 **	−0.209
Flow Experience→Innovative Behavior	0.651	0.079	8.237	***	0.564

Note: * *p* < 0.05, ** *p* < 0.01, *** *p* < 0.001.

## Data Availability

All data included in the current study can be obtained from the corresponding author through their email address upon reasonable request.
